# Commentary: Commonly Used Anesthesia/Euthanasia Methods for Brain Collection Differentially Impact MAPK Activity in Male and Female C57BL/6 Mice

**DOI:** 10.3389/fncel.2019.00219

**Published:** 2019-05-28

**Authors:** Samuel Kohtala, Tomi Rantamäki

**Affiliations:** ^1^Division of Pharmacology and Pharmacotherapy, Laboratory of Neurotherapeutics, Faculty of Pharmacy, University of Helsinki, Helsinki, Finland; ^2^SleepWell Research Program, Faculty of Medicine, University of Helsinki, Helsinki, Finland

**Keywords:** MAPK (mitogen-activated protein kinase), isoflurane, ketamine, anesthesia, carbon dioxide, nitrous oxide, ERK (extracellular-signal-regulated kinase), phosphorylation

A recent study in Frontiers in Cellular Neuroscience (Ko et al., 2019) investigated an important area of research of how different methods of anesthesia and euthanasia affect mitogen-activated protein kinase (MAPK) activity in various brain areas. The authors demonstrate significant changes in p44/42-MAPK (i.e., ERK1/2) phosphorylation after four different treatments: 45-min ketamine/xylazine anesthesia, 5-min CO_2_ euthanasia, 5-min isoflurane anesthesia, or decapitation. While the authors acknowledge the importance of MAPKs as targets of investigation, they fail to account for the temporal complexity of these signaling events and many previously published findings. Here we describe several lines of research that are important to consider for studying p44/42-MAPK signaling and address some of the limitations of the study.

We have investigated the regulation of p44/42-MAPK^Thr202/Tyr204^ phosphorylation with several anesthetics and brain areas (Kohtala et al., 2016, 2018). For example, we have shown MAPK phosphorylation to decrease in the adult male mouse hippocampus after a 30 min treatment with isoflurane (4% induction, 2% maintenance), sevoflurane (6% induction, 4.5% maintenance), urethane (2 g/kg, IP), and ketamine (100 mg/kg, IP) (Kohtala et al., 2016). Moreover, medetomidine, an α_2_-agonist, decreases MAPK phosphorylation in the adult mouse prefrontal cortex (PFC) during ongoing slow electroencephalogram (EEG) activity (Kohtala et al., 2018). The decrease in phosphorylation appears rapidly, since an anesthetic dose of ketamine (200 mg/kg, IP) reduced MAPK phosphorylation in the PFC within 3 min (Kohtala S, unpublished findings). Others have reported similar decreases with pentobarbital, ethanol, chloral hydrate and isoflurane (Salort et al., 2019).

All anesthetics we have investigated have similar effects on the phosphorylation of p44/42-MAPK^Thr202/Tyr204^, even though their pharmacological mechanisms vary. For example, the volatile anesthetics isoflurane and sevoflurane increase the activity of inhibitory gamma-aminobutyric acid A (GABA_A_) receptors and increase glycine-induced Cl^−^ currents unlike ketamine (Krasowski and Harrison, 1999). Ketamine has more potent effects on the inhibition of N-methyl-*d*-aspartate (NMDA) receptors (Yamakura and Harris, 2000). Urethane likely has the widest profile of pharmacological targets, increasing both glycine and GABA_A_ receptor function, while its inhibition of NMDA and α-amino-3-hydroxy-5-methyl-4-isoxazolepropionic acid (AMPA) receptors is considered more modest (Hara and Harris, 2002). Based on our previous studies, we have hypothesized that these anesthetics may cause some of their molecular alterations by having similar effects on the global brain state (i.e., anesthesia or sedation) despite their pharmacological differences (Kohtala et al., 2016, 2018).

To further complicate the interpretation of phosphorylation findings, we found a subanesthetic dose of ketamine (10 mg/kg, IP) or exposure to 50% nitrous oxide (N_2_O), another NMDAR antagonist, to increase p44/42-MAPK phosphorylation in the PFC of mice at 30 min (Kohtala et al., 2018). Similar findings of ketamine have been published by others. For example, Li et al. (2010) reported increased phosphorylation after 10 mg/kg ketamine, but not after 1, 5, or 80 mg/kg when measured from PFC synaptosomes at 1 h. Importantly, several lines of research link increases in p44/42-MAPK phosphorylation to enhanced cortical excitability. Electroconvulsive shock (ECS) has been demonstrated to increase MAPK phosphorylation in several different brain areas of rodents (Baraban et al., 1993; Bhat et al., 1998; Jeon et al., 1998; Kang et al., 2002; Yamagata et al., 2002; Hansen et al., 2007). Moreover, MAPK phosphorylation is also increased after glutamate administration to *ex vivo* brain slices (Vanhoutte et al., 1999). In line with these findings, subanesthetic doses of ketamine have been suggested to inhibit NMDARs present in GABAergic interneurons, decrease inhibition of excitatory pyramidal neurons and increase glutamatergic signaling (Moghaddam et al., 1997; Homayoun and Moghaddam, 2007). Indeed, the differential regulation of glutamate dynamics with small and large doses of ketamine (Moghaddam et al., 1997) remains a plausible explanation for the decrease in MAPK phosphorylation after high doses.

The results of Ko et al. suggest that CO_2_ asphyxiation produces the lowest level of MAPK activation and propose this method for brain tissue collection. The authors further consider the level of CO_2_ induced phosphorylation to be close to the physiological baseline and regard relative increases caused by other treatments as increased activation. However, phosphorylation changes are dynamic with both increases and decreases taking place in complex spatiotemporal patterns. In stark contrast with the authors conclusions, we interpret these results to demonstrate that CO_2_ produces a pronounced decrease in MAPK phosphorylation, thus presenting a clear bias for biochemical analyses.

Ko et al. argue that the increased phosphorylation after 5% isoflurane may mimic a 30 min treatment with 0.7% isoflurane, since this has been shown to increase MAPK activity in a previous study (Liu et al., 2014). We disagree, as we have previously validated the effects of isoflurane anesthesia in mice using EEG and found 4% isoflurane to lead into an EEG burst-suppression pattern within minutes (Kohtala et al., 2016; Theilmann et al., 2019). Indeed, 5% isoflurane or CO_2_ inhalation is likely to result in rapid isoelectric EEG and eventually death.

Surprisingly, Ko et al. state that their findings are in agreement with a study by Overmyer et al. (2015), who investigated the effects of anesthesia/euthanasia on the metabolomics of peripheral tissues in mice. Overmyer et al. state that CO_2_ and isoflurane overdose resulted in death or respiratory cessation within 2.5 min and suggest that metabolomic changes may take place rapidly after the cessation of circulation. The conclusions of Overmyer et al. explicitly state that hypoxia induced by euthanasia produces the most rapid and dramatic alterations in the metabolome. Notably, a rapid loss of physiological or anesthesia induced phosphorylation has been shown to occur in brain samples collected 2 min post-mortem, highlighting the importance of rapid dissection (Li et al., 2005). This leaves open the question whether the animals in the study by Ko et al. had stopped breathing before the collection of brain tissue.

Several technical questions were raised by the study. First, Ko et al. compared four groups with significantly different treatment time points, which complicate the interpretation of the findings. The authors did not disclose whether heating was applied during the treatments. Anesthesia induced hypothermia has been shown to reduce p44/42-MAPK phosphorylation in rats (Whittington et al., 2013). Since treatment lengths varied from 5 to 45 min, large differences in temperature regulation may have taken place.

Second, Ko et al. suggest that higher MAPK activity in decapitated animals compared to CO_2_ asphyxiation may be due to stress. We argue that 100% CO_2_ asphyxiation is likely more stressful to the animals, since it has been shown to cause pain and aversion (Niel et al., 2008), and that brief restraint before decapitation is unlikely to have a significant stress effect. In addition, the authors did not consider the effects of injection stress, since the decapitation, CO_2_ and isoflurane groups were not treated with a saline injection.

Third, the methods had several minor inconsistencies. The authors left unclear whether they only used a protease inhibitor in sample lysis buffer. The use of phosphatase inhibitors is warranted when measuring phosphorylation changes. Moreover, the authors describe having an *n* = 3 for brain regions other than the dorsal striatum and hippocampus, which had *n* = 6. It remains unclear how the number of samples was doubled, but it can be assumed that bilateral samples from the same animals were collected. Multiple samples from the same animal do not represent biological replicates.

In conclusion, the choice of method for anesthesia, euthanasia and sample collection may significantly affect molecular outcomes. Decapitation, or perhaps microwave irradiation (Fernandes and Li, 2017), remains the most optimal choice for animal experiments where protein phosphorylation is investigated (Kohtala et al., 2016).

## Author Contributions

SK wrote the first draft of the manuscript. TR contributed ideas and writing into the generation of the final manuscript.

### Conflict of Interest Statement

The authors declare that the research was conducted in the absence of any commercial or financial relationships that could be construed as a potential conflict of interest.
